# KRAS Withdrawal in Cholangiocarcinoma Leads to Immune Infiltration and Tumor Regression

**DOI:** 10.1002/advs.202511312

**Published:** 2025-12-03

**Authors:** Youwei Qiao, Matthew F. Yee, Chaitanya N. Parikh, Boyang Ma, Yueying Cao, Yu‐Huan Shih, Joae Qiong Wu, Marcus Ruscetti, Shun‐Qing Liang, Wen Xue

**Affiliations:** ^1^ RNA Therapeutics Institute University of Massachusetts Chan Medical School Worcester MA 01605 USA; ^2^ Department of Molecular Cell and Cancer Biology University of Massachusetts Chan Medical School Worcester MA 01605 USA; ^3^ Biochemistry and Molecular Biotechnology University of Massachusetts Chan Medical School Worcester MA 01605 USA; ^4^ Department of Medicine University of Minnesota Twin Cities Minneapolis MN 55455 USA; ^5^ Department of Molecular Medicine University of Massachusetts Chan Medical School Worcester MA 01605 USA; ^6^ Li Weibo Institute for Rare Diseases Research University of Massachusetts Chan Medical School Worcester MA 01605 USA

**Keywords:** biliary malignancy, cholangiocarcinoma, KRAS inhibition, oncology

## Abstract

Cholangiocarcinoma (CCA) is a liver cancer subtype with poor survival rates. *KRAS* mutations are found in 15–40% of CCA, representing a new potential treatment target. Whether KRAS inhibition leads to CCA tumor regression is unknown, partly due to the lack of conditional animal models. A conditional TRE.*Kras* ^G12D^/*Trp53* knock‐out (TKP) CCA mouse model is engineered using the transposon system and CRISPR‐Cas9. Withdrawal of *Kras* ^G12D^ results in >90% tumor regression by day 7, accompanied by infiltration and enrichment of activated CD8^+^ T cells, shown by IHC, co‐IF staining, and single‐cell RNA‐Seq. Bulk RNA‐Seq of TKP cell line suggested that *Kras* ^G12D^ withdrawal stimulates the transforming growth factor beta pathway and induces senescence. Cytokine array characterizes the secretion of pro‐inflammatory factors, including IL‐15 and CCL17. Lentiviral overexpression of murine IL‐15 and CCL17 delays CCA tumor progression in a syngeneic transplant model. Consistently, expression of IL‐15 resulted in blockade of tumor progression in the TKP CCA model. These findings highlight the importance of oncogenic *Kras* in CCA tumor maintenance and underscore KRAS inhibition as a potential therapeutic approach for CCA.

## Graphical abstract

Cholangiocarcinoma (CCA) driven by oncogenic KRAS depends on its continuous activation for tumor maintenance. Using a conditional KRAS model, the authors show that turning off KRAS triggers rapid tumor regression accompanied by immune cell infiltration and cytokine release. The findings uncover a KRAS–senescence–immune signaling axis and highlight KRAS inhibition as a promising therapeutic strategy for CCA.

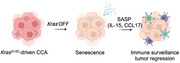

## 1 Introduction

Cholangiocarcinoma (CCA) is the second most prevalent type of primary liver cancer, which accounts for roughly 15% liver tumors.^[^ ^1^, ^2^, ^3^ ^]^ Currently, surgical resection is the preferred treatment for CCA,^[^ ^1^, ^2^, ^3^ ^]^ but most patients are diagnosed at an advanced stage with unresectable disease, due to a lack of tumor markers for early diagnosis. The prognosis of CCA is therefore dismal, with a 5‐year survival rate of 10%.^[^ ^1^, ^2^, ^3^ ^]^ Targeted molecular therapies for CCA are in clinical trials, including isocitrate dehydrogenase 1 (IDH1) inhibitors and fibroblast growth factor receptor 2 (FGFR2) inhibitors.^[^ ^1^, ^4^, ^5^ ^]^ However, these targeted molecular therapies can only benefit a small percentage of patients due to the low prevalence of IDH1 and FGFR2 mutations in CCA. Thus, there is an urgent need to develop novel targeted molecular therapies that benefit more CCA patients.

*KRAS* is one of the most frequently mutated proto‐oncogenes in CCA. Fifteen to forty percent of patients with CCA harbor a *KRAS* mutation.^[^ ^1^, ^2^, ^3^, ^6^, ^7^, ^8^ ^]^ KRAS was regarded as an “undruggable” target for decades due to the lack of binding pockets for small molecule inhibitors on its “smooth” surface.^[^ ^7^, ^9^ ^]^ Recently, several selective, potent inhibitors for mutant KRAS have been developed, including KRAS(G12C) inhibitor “sotorasib”, KRAS(G12D) inhibitor MRTX1133, and, most recently, the RAS(ON) multi‐selective pan‐KRAS inhibitor RMC‐6236.^[^ ^10^, ^11^, ^12^, ^13^ ^]^ Several preclinical studies have shown potent tumor growth inhibition of KRAS inhibitors in KRAS‐driven pancreatic ductal adenocarcinoma (PDAC), colon cancer, and lung cancer mouse models.^[^ ^13^, ^14^ ^]^ Furthermore, KRAS inhibitors, including sotorasib (NCT04933695) and RMC‐6236 (NCT05379985), are being tested in the clinic for metastatic, unresectable KRAS‐driven cancers such as PDAC and non‐small cell lung cancer treatment.^[^ ^15^ ^]^ Therefore, targeting *KRAS* mutations has become a potential therapeutic approach for CCA patients with advanced disease. However, there are limited in vivo models to study the mechanism by which *KRAS* mutations promote tumor maintenance in CCA. Thus, it is crucial to conduct animal studies to explore the therapeutic potential of KRAS inhibition for CCA treatment.

*KRAS* is a member of the rat sarcoma viral oncogenes (RAS) family, which encodes a GTPase that functions as a molecular switch regulating intracellular signaling cascades by switching between activated and inactivate conformations.^[^ ^10^, ^11^, ^16^ ^]^ Oncogenic mutations result in a constitutively activated KRAS and contribute to cancer cell proliferation, tumor metabolism, and resistance to cell death and cell cycle arrest.^[^ ^17^, ^18^, ^19^ ^]^ A previous study has shown that oncogenic *Kras* withdrawal induces apoptotic cell death in an inducible *Kras*‐driven lung adenocarcinoma mouse model and PDAC models.^[^ ^20^, ^21^, ^22^ ^]^ However, many questions remain poorly understood, including whether KRAS inhibition leads to CCA tumor regression, what the mechanisms of tumor regression are, and what role the immune system plays in this process.

In this study, we investigate the role of oncogenic *Kras* in tumor maintenance in an inducible *Kras*‐driven CCA mouse model. We develop a conditional CCA mouse model by codelivery of plasmids encoding a transposon‐borne inducible *Kras* ^G12D^ transgene and Cas9 and *p53* guide RNA. We show that *Kras*‐driven CCA tumors regress upon withdrawal of oncogenic *Kras*, accompanied by activation of the transforming growth factor beta pathway and cellular senescence. Senescent CCA tumor cells secrete pro‐inflammatory factors, including IL‐15 and CCL17, and lentiviral overexpression of either IL‐15 or CCL17 delays Ras‐driven CCA xenograft tumor progression. Consistent with results in the transplant model, expression of IL‐15 delays tumor progression in our TKP CCA model.

## 2 Results

### 2.1 Conditional Overexpression of *Kras* ^G12D^ with Trp53 Knockout Drives the Formation of Cholangiocarcinoma in C57BL/6 Mice

**Figure 1:**
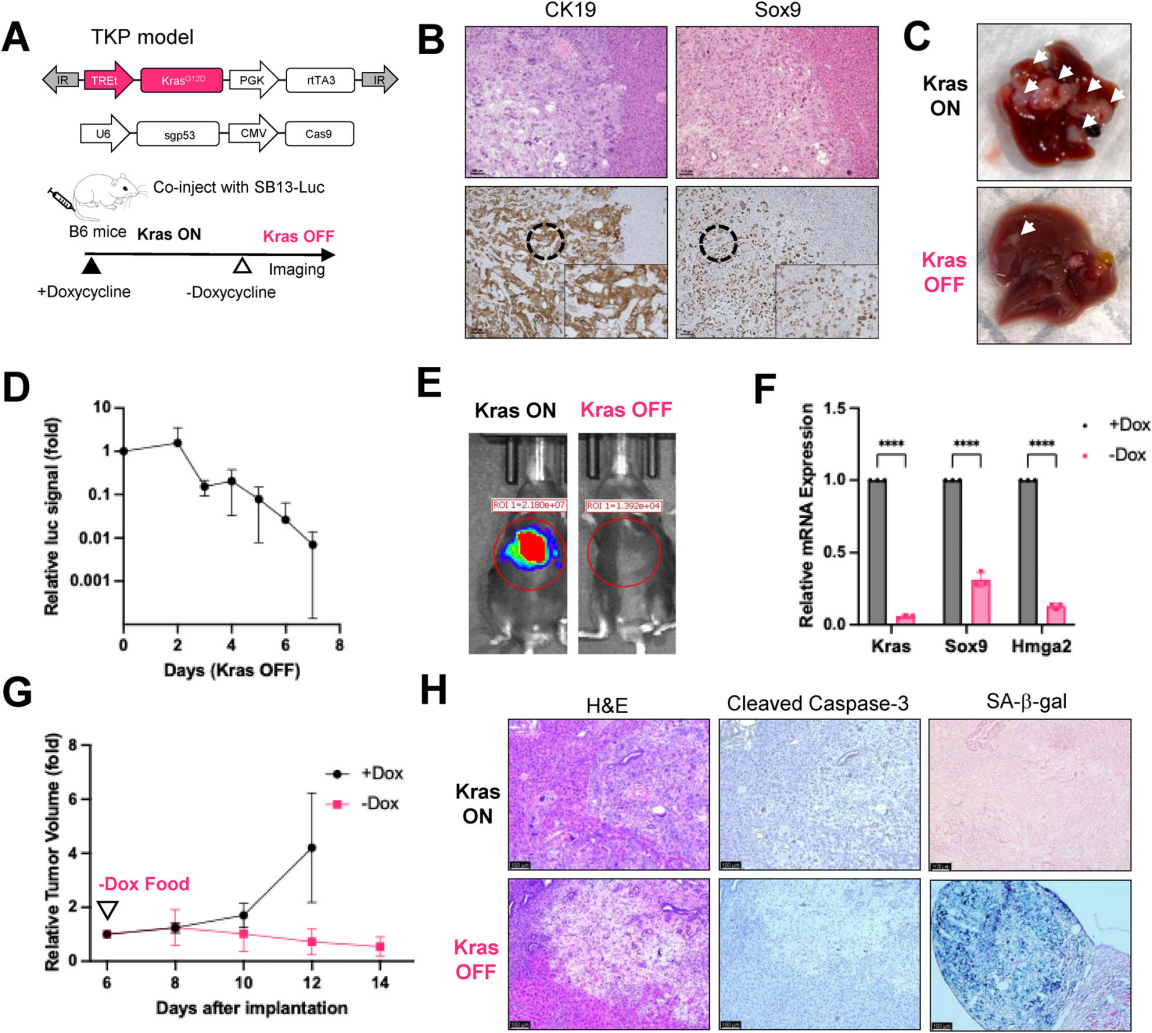
Oncogenic *Kras* ^G12D^ withdrawal in the CCA mouse model induces tumor regression and senescence. A) Generating a doxycycline‐inducible CCA mouse model using *Sleeping Beauty* transposon and CRISPR/Cas9 (TRE.*Kras* ^G12D^;*Trp53* KO, TKP model). Plasmids encoding transposase with luciferase reporter, TRE.Kras^G12D^ transposon, and Cas9 with sgRNA targeting *Trp53* were hydrodynamically injected into immunocompetent B6 mice. B) Representative Hematoxylin & eosin (H&E) and immunohistochemistry (IHC) staining of Kras ON tumors 4 weeks after injection. CK19, Cytokeratin 19. Sox9, SRY‐Box Transcription Factor 9. Scale bar=100 µm. C) Representative gross liver images of TKP *Kras* ON and TKP *Kras* OFF mice. Arrows indicate tumors. D) Relative luciferase signal of liver tumors demonstrates tumor regression. Day 0 (*Kras*, ON) is set as 1. Data are presented as mean ± SD. (n=3). E) Representative luciferase imaging shows *Kras* OFF tumor regression. F) qRT‐PCR analysis of *Kras*, *Sox9*, and *Hmga2* mRNA expression in TKP cell line on Dox or off Dox at day 8. Each group has three biological replicates. Data are presented as mean ± SD. P‐values were calculated by an unpaired t‐test. ^****^ *P* <0.0001. G) Change in tumor volume of mice bearing subcutaneous TKP tumors. Tumor volume at day 6 is set as 1. Data are presented as mean ± SD. (n=4 for +Dox group and n=6 for ‐Dox group). H) Representative H&E staining and IHC staining, and senescence‐associated beta‐galactosidase (SA‐β‐Gal) staining of TKP *Kras* ON and TKP *Kras* OFF tumors. *Kras* OFF tumors are positive for SA‐β‐Gal and negative for Cleaved Caspase‐3. Scale bars are 100 µm.

A cancer genomic study of 195 patients suggests that *KRAS* and *TP53* are among the most frequently altered genes in cholangiocarcinoma (CCA)^[^ ^7^ ^]^ (Figure S1A, Supporting Information). According to this study, thirty‐one percent of patients harbor *TP53* mutations, and sixteen percent of patients harbor *KRAS* mutations (Figure S1B, Supporting Information). Among CCA patients who harbor *KRAS* mutations, over forty percent carry *KRAS* ^G12D^ mutations^[^ ^23^ ^]^ (Figure S1C, Supporting Information). In mice, liver‐specific expression of *Kras* ^G12D^ with deletion of *Trp53* gene drives the formation of liver tumors that resemble CCA both histologically and molecularly—e.g., activation of MAPK/ERK and PI3K/AKT pathways.^[^ ^24^, ^25^, ^26^ ^]^ To engineer an inducible model of CCA, we delivered a Sleeping Beauty transposon plasmid encoding Tet‐On‐inducible *Kras* ^G12D^ transgene and reverse‐tetracycline transactivator (rtTA), a plasmid encoding Cas9 nuclease and *Trp53* guide RNA,^[^ ^27^, ^28^, ^29^ ^]^ and a plasmid encoding Sleeping Beauty transposase and luciferase reporter to the mouse liver by hydrodynamic tail‐vein injection (**Figure** **1**A). Expression of oncogenic *Kras* ^G12D^ was induced by feeding mice a doxycycline diet to promote tumor progression (*Kras* ON). To investigate the effect of oncogenic *Kras* ^G12D^ withdrawal in the CCA tumor, the doxycycline diet was replaced with a normal rodent diet (*Kras* OFF) (Figure 1A). We named this mouse model TRE.*Kras* ^G12D^/*p53* knockout CCA (TKP).

Consistent with a previous study using constitutive expression of *Kras* ^G12D^,^[^ ^26^ ^]^ inducible overexpression of *Kras* ^G12D^ with p53 knockout drove the formation of liver tumors that were CK19‐positive, a key diagnostic marker of CCA^[^ ^30^ ^]^ (Figure 1B). The tumor additionally showed elevated Sox9 expression by IHC, a transcription factor linked to poor prognosis in human CCA, highlighting the aggressiveness of our tumor model^[^ ^31^ ^]^ (Figure 1B). The earliest visible surface tumors appeared around 2 weeks post‐injection; luciferase signal could be detected at 3 weeks; and at 4 weeks post‐injection, *Kras* ON mice developed extensive liver tumors (Figure 1C, ***Kras* ON**), and were hunched and sick. The majority of tumor‐bearing mice succumbed within six weeks post‐injection. These findings suggested that our tumor mouse model resembles aggressive human CCA tumors with poor prognosis.

### 2.2 Oncogenic *Kras* ^G12D^ Withdrawal Results in Cellular Senescence and Tumor Regression In Vivo

The TKP model allowed us to study the effects of *Kras* ^G12D^ withdrawal. To investigate whether *Kras* ^G12D^ withdrawal leads to tumor regression in the inducible CCA mouse model, we removed the doxycycline diet at ≈4 weeks post‐injection (Figure 1A). Bioluminescent imaging of mice revealed a steady decline in tumor size to an ≈99% reduction by day 7 (Figure 1D,E; Figure S2A, Supporting Information). Surface liver tumors were therefore significantly reduced at 8 days after doxycycline withdrawal (Figure 1C, *Kras* OFF). Quantitative analysis confirmed that tumor weights were markedly decreased after doxycycline withdrawal at day 5, consistent with the bioluminescent imaging and gross observations (Figure S2B, Supporting Information). To confirm the conditional expression of *Kras*, we isolated the TKP cell line from a *Kras* ON solid tumor 4 weeks post‐injection and maintained the TKP cell line in medium with doxycycline (+Dox). Consistent with previous studies showing high expression level of HMGA2 is associated with poor survival in liver cancer patients,^[^ ^32^ ^]^ *Kras* ON cells expressed high levels of liver progenitor cell markers Hmga2 and Sox9 (Figure 1F). Oncogenic *Kras* withdrawal by removing doxycycline (‐Dox) resulted in a dramatic downregulation of *Kras*, *Sox9*, and *Hmga2* expression in the TKP cell line (Figure 1F). To test whether tumors regress upon *Kras* withdrawal in a transplant model, we injected the TKP cell line subcutaneously into B6 mice fed on a doxycycline diet, and after tumor onset (6 days post‐injection), we removed doxycycline from the diet. Whereas xenograft tumors continued to grow in mice that remained on doxycycline, xenograft tumors shrank in mice taken off doxycycline, validating that oncogenic *Kras* withdrawal results in tumor regression (Figure 1G).

Oncogenic *KRAS* plays a vital role in regulating cell death and cell cycle arrest mechanisms, promoting cancer cell proliferation.^[^ ^17^, ^18^, ^19^ ^]^ To determine if tumor regression results from cell death or cell cycle arrest, we performed IHC staining on *Kras* ON tumors and day 8 *Kras* OFF tumors. *Kras* OFF tumors were negative for Cleaved‐Caspase 3, indicating that apoptotic cell death is not the mechanism of tumor regression upon Kras withdrawal (Figure 1H). *Kras* OFF tumors were positive for senescence‐associated beta‐galactosidase (SA‐β‐gal) activity (Figure 1H), suggesting that oncogenic *Kras* withdrawal results in cellular senescence.

**Figure 2:**
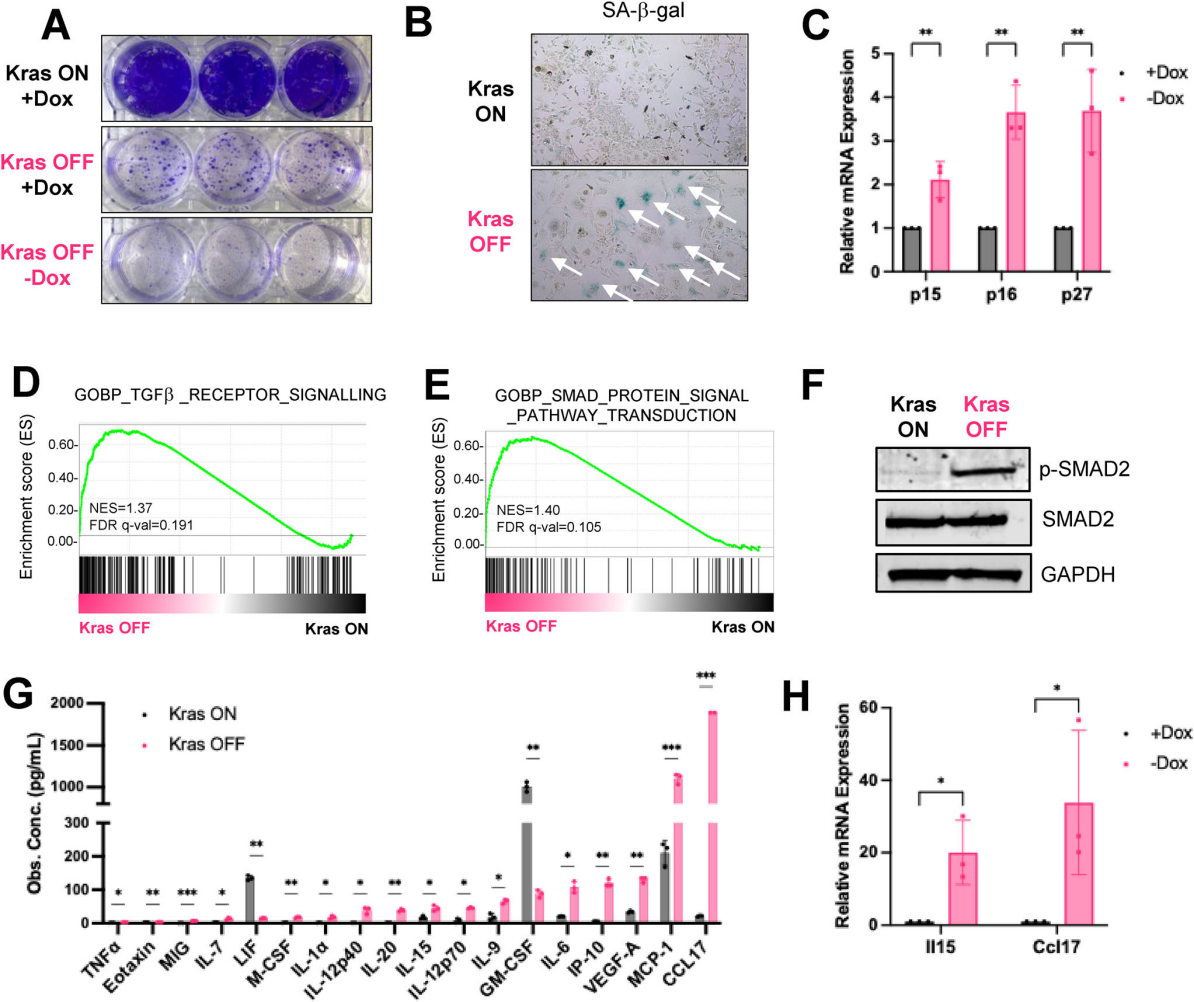
*Kras* ^G12D^ withdrawal results in cellular senescence and alters the secretory phenotype. A) Colony formation assay of TKP *Kras* ON and *Kras* OFF cells. *Kras* OFF (+Dox) TKP cells indicated *Kras* OFF TKP cells, which were re‐introduced with doxycycline. Each group has three technical replicates. B) Representative SA‐β‐Gal staining of TKP *Kras* ON and *Kras* OFF cells. Arrows indicate senescent cells. C) qRT‐PCR analysis of p15, p16, and p27 mRNA expression in TKP cell line on Dox or off Dox at day 8. Each group has three biological replicates. D, E) GSEA analysis in TKP *Kras* OFF and *Kras* ON cell lines. NES, normalized enrichment score. FDR, false discovery rate. F) Phospho‐SMAD2, SMAD2, and GAPDH western blot in TKP *Kras* ON and *Kras* OFF cell line. Cells were starved with 0.2% FBS. G) Cytokine array of TKP *Kras* ON and *Kras* OFF cell line. Each group has three biological replicates. One outlier from the CCL17 group is omitted. H) qRT‐PCR analysis of Il15 and Ccl17 in Kras ON and Kras OFF TKP cell line. Each group has three biological replicates. (B, H) Data are presented as mean ± SD. P‐values were calculated by an unpaired t‐test. (G) Data are presented as mean ± SD. P‐values were calculated by a paired t‐test. ^*^ *P* <0.05, ^**^ *P* <0.01, ^***^ *P* <0.001, ^****^ *P* <0.0001.

TKP cells derived from solid tumors exhibited robust growth and rapid proliferation under doxycycline treatment (**Figure** **2**A, ***Kras* ON +Dox**). TKP cells showed abrogated cell growth upon *Kras* ^G12D^ withdrawal, and replated *Kras* OFF TKP cells failed to proliferate without doxycycline (Figure 2A, ***Kras* OFF ‐Dox**). Also, the replated *Kras* OFF TKP cells exhibited minimal growth even with the addition of doxycycline and reactivation of *Kras* ^G12D^ expression, suggesting that TKP cells may undergo cell cycle arrest following *Kras* withdrawal (Figure 2A, ***Kras* OFF +Dox**). *Kras* OFF TKP cells were positive for SA‐β‐gal activity and exhibited a significantly altered morphology compared to proliferating *Kras* ON cells, characterized by an enlarged, flattened appearance with expanded nuclei (Figure 2B). Additional molecular markers of cellular senescence—p15, p16, and p27—were upregulated in *Kras* OFF TKP cells compared to *Kras* ON TKP cells (Figure 2C), indicating that oncogenic *Kras* withdrawal results in cell cycle arrest and cellular senescence.

### 2.3 *Kras* ^G12D^ Withdrawal Changes the Transcriptional Landscape of TKP CCA Cell Line

RNA‐Seq analyses revealed that *Kras* ^G12D^ withdrawal significantly altered the transcriptional landscape of TKP CCA cells (Figure S3A, Supporting Information). Pearson correlation analysis between RNA‐Seq replicates revealed strong positive correlations, supporting the reliability of the datasets for differential expression analyses (Figure S3B, Supporting Information). As expected, *Kras* transcript levels (reads‐per‐kilobase per million mapped reads; RPKM) were significantly reduced by ≈5‐fold in *Kras* OFF TKP cells (Figure S3C, Supporting Information). Among all differentially expressed genes, we identified 1,430 genes significantly upregulated and 644 genes significantly downregulated (≥2‐fold; Figure S3D, Supporting Information). Interestingly, genes downregulated upon *Kras* ^G12D^ withdrawal included *Lilr4b* and *Cxcr2* ^[^ ^33^, ^34^, ^35^ ^]^ (Figure S3A, Supporting Information). Blockade of CXCR2 has been shown to promote anti‐tumor immunity, suggesting that *Kras* ^G12D^ withdrawal might trigger an immune response.^[^ ^36^, ^37^ ^]^ Gene set enrichment analysis (GSEA) suggested that pathways related to transforming growth factor beta (TGF‐β) signaling (Figure 2D; Figure S4A, Supporting Information) and SMAD protein signaling (Figure 2E; Figure S4B–D, Supporting Information) were among the top‐enriched gene sets upon *Kras* ^G12D^ withdrawal in TKP cells. Indeed, *Tgfb2* levels were markedly upregulated in the RNA‐seq data (Figure S3A, Supporting Information). Consistent with the upregulation of TGF‐β–SMAD signaling, we detected increased levels of phosphorylated SMAD2 (p‐SMAD2) in *Kras* OFF TKP cells compared to *Kras* ON TKP cells, whereas total SMAD2 protein levels were the same (Figure 2F). To further validate that the activation of TGF‐β pathway in vivo, we performed single‐cell RNA sequencing (scRNA‐Seq) of TKP *Kras* ON and OFF tumors. Pathway enrichment analysis identified TGF‐β pathway as one of the most activated pathways across several different clusters, including C4, identified as the T cell cluster, and C9/13, identified as the tumor cell cluster (Figure S7A, Supporting Information). A previous study has shown that treating liver cancer cells with TGF‐β activates SMAD protein signaling pathway, upregulates cell cycle inhibitors p15 and p21, and induces cellular senescence.^[^ ^38^ ^]^ Similarly, our results showed that oncogenic *Kras* withdrawal inhibited TKP cell proliferation, activated TGF‐β and SMAD signaling pathway, and induced cellular senescence.

### 2.4 Oncogenic *Kras* ^G12D^ Withdrawal Alters the Secretory Phenotype of TKP CCA Cell Line

Cellular senescence arrests tumor growth by downregulating proliferation genes and upregulating genes encoding anti‐tumorigenic secretory factors known as senescence‐associated secretory phenotype (SASP).^[^ ^39^, ^40^, ^41^, ^42^ ^]^ SASP factors often include proinflammatory chemokines and cytokines that recruit and activate tumor‐fighting immune cells, angiogenic factors, growth factors, and matrix metalloproteinases.^[^ ^39^, ^40^, ^41^, ^42^ ^]^ Bulk RNA‐Seq analysis of TKP cell line revealed that *Kras* ^G12D^ withdrawal altered the transcription level of essential cell cycle and senescence‐associated secretory phenotype (SASP) genes, indicating that oncogenic *Kras* withdrawal induces cell cycle arrest and a pro‐inflammatory, senescent phenotype (Figure S3E,F, Supporting Information). Consistent with the bulk RNA‐Seq results, cytokine array analyses of *Kras* OFF TKP cells and *Kras* ON TKP cells revealed that *Kras* ^G12D^ withdrawal stimulates the secretions of SASP factors, including those involved in NK cell recruitment (IP‐10/CXCL10 and MCP‐1/CCL2), cytokines involved in CD8^+^ T cell and NK cell activation and proliferation (IL‐12, IL‐15, TNF‐α), and angiogenic factors (VEGF)^[^ ^40^, ^41^ ^]^ (Figure 2G; Figure S5A, Supporting Information). A previous study has shown that IL‐15 can suppress metastatic and primary liver cancer by activation of CD8^+^ T cells.^[^ ^43^ ^]^ Interestingly, CCL17, a chemokine that is associated with poor survival in hepatocellular carcinoma (HCC) patients, increased dramatically upon *Kras* ^G12D^ withdrawal in TKP cells^[^ ^44^, ^45^, ^46^ ^]^ (Figure 2G; Figure S5A, Supporting Information). IL15 and CCL17 were significantly upregulated upon *Kras* ^G12D^ withdrawal in our RNA‐Seq analyses and by RT‐qPCR (Figure 2H; Figure S3A, Supporting Information). These results indicated that *Kras* ^G12D^ withdrawal in TKP cells induced secretion of SASP factors, including a well‐studied cytokine in liver cancer (IL‐15) and a less well‐characterized chemokine (CCL17).

Previous studies have shown that SASP involves activation of the NF‐kB pathway, which can induce expression of IL‐15 and CCL17.^[^ ^47^ ^]^ Our GSEA results further indicated enrichment of pathways associated with inflammatory and immune signaling upon *Kras* ^G12D^ withdrawal, including interferon alpha and gamma pathways (Figure S5B,C, Supporting Information). To confirm these observations, we performed qRT‐PCR analysis and observed increased mRNA levels of IL‐15 when we treated TKP *Kras* ON cells with interferon alpha and gamma (Figure S5D,E, Supporting Information). These results supported our hypothesis that IL‐15 is regulated by interferon signaling pathways.

### 2.5 *Kras* ^G12D^ Withdrawal Alters Cellular Subpopulations and Recruits CD8 T Cells to Tumors

**Figure 3:**
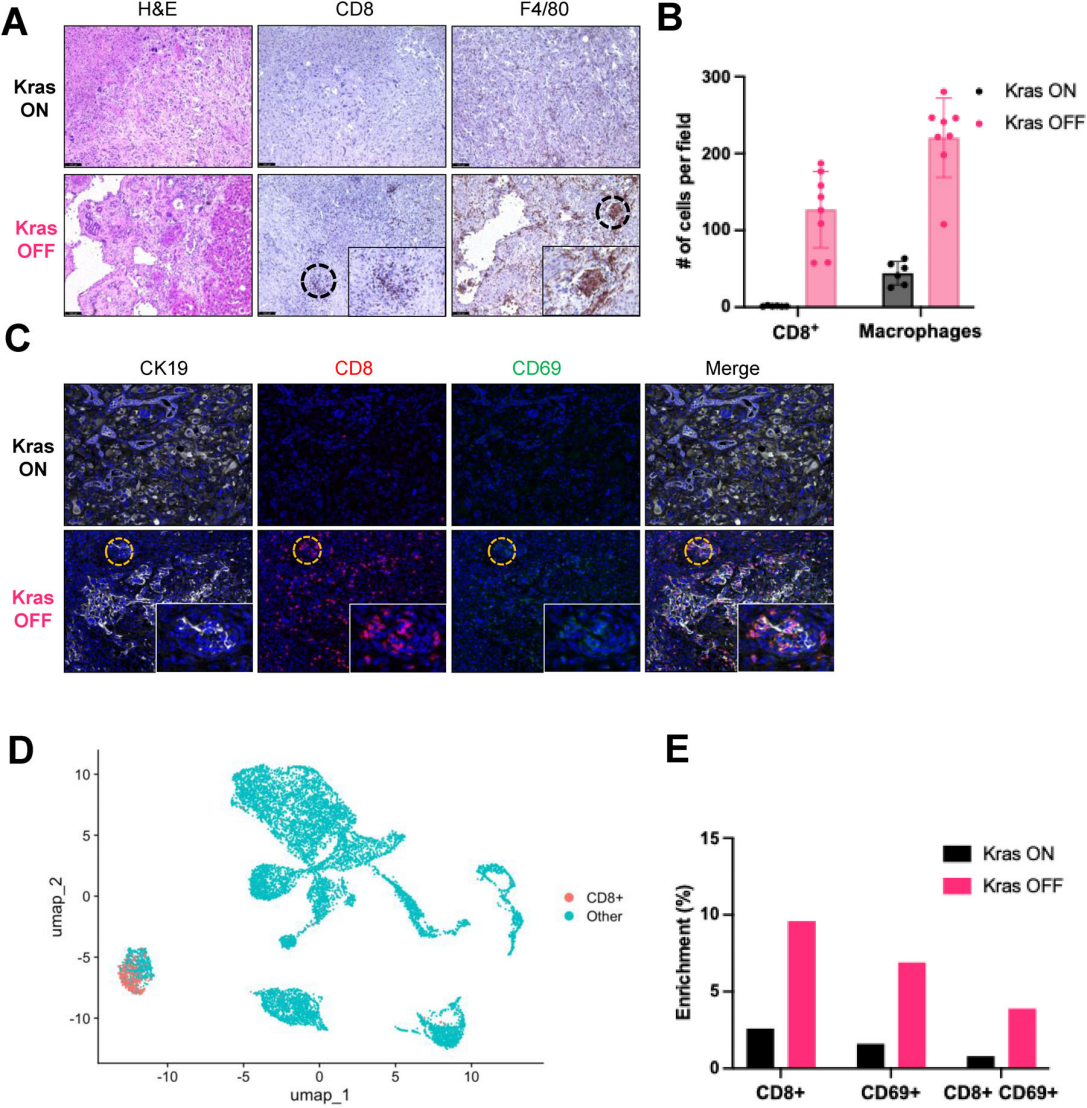
*Kras* ^G12D^ withdrawal recruits CD8 T cells and Macrophages to the tumor. A) H&E and IHC staining of TKP *Kras* ON and *Kras* OFF tumors. *Kras* OFF tumors were collected 8 days after doxycycline withdrawal. B) Quantification of CD8 and F4/80 in TKP *Kras* ON tumors (n=6) and *Kras* OFF tumors (n=8). Each data point represents the mean of positive cells of ten 20X microscopic fields. Lines represent the mean value. P‐values were calculated by an unpaired t‐test. ***P <0.001. C) Representative immunofluorescence images of CK19, CD8, and CD69 positive cells in TKP *Kras* ON and *Kras* OFF tumors. D) UMAP visualization of CD8^+^ T cell clusters identified by scRNA‐seq of 13,329 cells from one TKP *Kras* ON and one TKP *Kras* OFF tumor. E) Bar graph indicating the enrichment of CD8^+^ T cells, CD69^+^ T cells, and CD8^+^ CD69^+^ T cells in TKP *Kras* ON and *Kras* OFF tumors by scRNA‐seq.

We next explored whether SASP induction triggered by oncogenic *Kras* withdrawal activates CD8^+^ T cells. In histological analyses of *Kras* ON tumors and day 8 *Kras* OFF tumors, we detected a dramatic increase in CD8^+^ T cells and F4/80^+^ macrophages in *Kras* OFF tumors compared to *Kras* ON tumors (**Figure** **3**A,B), suggesting that *Kras* ^G12D^ withdrawal from CCA tumors promotes an immune response. Immunofluorescence analysis of CK19, CD8, and activation marker CD69 revealed notable differences in T cell distribution and activation state in *Kras* ON and *Kras* OFF TKP tumors. In *Kras* ON tumors, CD8 T cells were sparsely distributed in CK19‐positive CCA tumor cells (Figure 3C, ***Kras* ON**). However, in *Kras* OFF tumors, there was a marked increase in the density of CD8 and CD69 double‐positive T cells clustering around CK19‐positive tumor cells (Figure 3C, ***Kras* OFF**).

To investigate the cellular diversity within TKP tumors and the effects of *Kras* ^G12D^ withdrawal, we performed scRNA‐seq on *Kras* ON and *Kras* OFF TKP tumors. We obtained transcriptomic profiles from a total of 13 329 cells, which were assigned to 15 distinct subclusters using UMAP dimensionality reduction and Louvain clustering (Figure S6A,B, Supporting Information). Cell types were identified based on the expression of established marker genes, including *Ptprc* for immune cells, *Csf1r*, *C1qa*, *Cd68*, and *Ccr2* for macrophages, *S100a8* and *Cxcr2* for neutrophils, *Cd3d* and *Cd3e* for T cells, *Pecam1* and *Flt1* for vascular cells, *Xcr1* and *Cd209a* for dendritic cells, *Krt8*, *Krt18*, and *rtTA3* for tumor cells, *Dcn*, *Col1a1*, and *Acta2* for cancer‐associated fibroblasts (CAFs), and *Cd79a*, *Cd19*, and *Igkc* for B cells (Figure S6C, Supporting Information).

*Kras* ^G12D^ withdrawal significantly altered the cellular composition of the tumor microenvironment. Specifically, T cells, B cells, vascular cells, and CAFs were significantly enriched in *Kras* OFF tumors compared to *Kras* ON tumors (Figure S6D,E, Supporting Information). To further explore the T cell compartment, we re‐clustered the T cell population and identified subpopulations of CD8^+^ T cells (expressing CD8a) and activated T cells (expressing CD69). Our analysis revealed that CD8^+^ T cells, CD69^+^ T cells, and double‐positive CD8^+^ CD69^+^ T cells were significantly more abundant in *Kras* OFF tumors (Figure 3D,E). This observation was corroborated by co‐immunofluorescence (co‐IF) staining, which similarly showed increased infiltration of CD8^+^ and CD69^+^ cells in *Kras* OFF tumors (Figure 3C). Moreover, pathway enrichment analysis from scRNA‐Seq data revealed a robust activation of interferon gamma (IFNγ) pathway in T cell cluster (cluster 4). L‐R interaction analysis further demonstrated elevated IL‐15‐related L‐R interaction scores across all clusters in TKP *Kras* OFF tumor (Figure S7A,B, Supporting Information). In particular, we found a strong *IL‐15‐IL‐2rb* interaction between the T cell cluster (cluster 4) and tumor cell cluster (cluster 13), suggesting potent T cell activation mediated through the IL‐15‐axis (Figure S7C, Supporting Information). These findings not only indicate that T cell response drives anti‐tumor immune response in TKP *Kras* OFF tumor, but also suggest that IL‐15 signaling may play a key role in promoting T cell activation.

To examine immune populations other than T cells, we analyzed NK cell subclusters identified within cluster 4 of the single‐cell dataset based on expression of the marker gene *Ncr1* (Figure S8A,C, Supporting Information). The analysis showed an increase in NK cell numbers in *Kras* OFF tumors, although their overall abundance in the tumor microenvironment remained low (Figure S8B, Supporting Information). Consistent with the scRNA‐seq results, IHC from three independent biological replicates also revealed a slight increase in NK cell staining in *Kras* OFF tumors, although the abundance of infiltrated NK cells was low (Figure S8D,E, Supporting Information). Together with the CD8⁺ T cell IHC, co‐IF, and single‐cell analyses, these data suggest that NK cells may participate to a limited extent, whereas CD8⁺ T cells are the main drivers of the immune changes following oncogenic *Kras* withdrawal.

### 2.6 Lentiviral Expression of Mouse IL‐15 or CCL17 Recruits and Activates CD8 T Cells to Delay Tumor Progression

**Figure 4:**
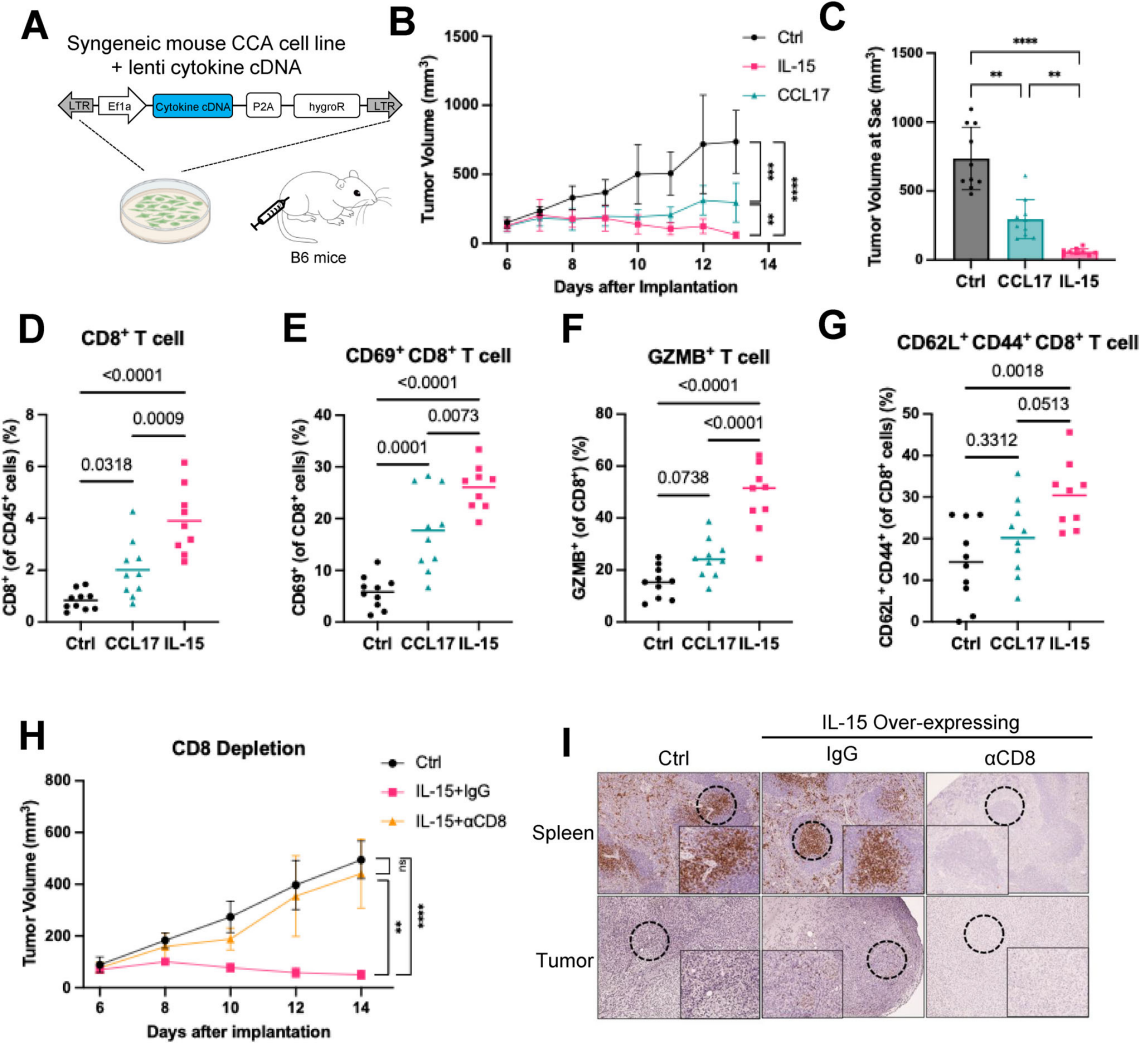
Murine IL‐15 or CCL17 delays the progression of syngeneic mouse CCA xenograft tumors. A) RIL‐175 CCA cell lines from a B6 genetic background were transduced with a lentiviral vector encoding a constitutive Ef1α promoter and mouse cytokine/chemokine cDNAs or an empty lentiviral vector as a control. Xenograft models were made by injecting 10^6^ cells subcutaneously into immunocompetent B6 mice. B) Overexpression of either IL‐15 or CCL17 delayed tumor progression. Control, n=10; IL‐15‐O/E, n=9, CCL17‐O/E, n=10. Data are presented as mean ± SD. C) Tumor volume at day 13 after injection. Data are presented as mean ± SD. D–G) Flow cytometry analysis in control, IL‐15‐overexpressed, and CCL17‐overexpressed tumors at day 13 after injection. Lines represent the mean value. E) CD69, an activated T cell marker. (F) GZMB, granzyme B. (G) CD62L^+^ CD44^+^, memory T cells markers. H) CD8⁺ T cell depletion abolishes the anti‐tumor effect induced by IL‐15. Tumor growth curves of control and IL‐15 overexpressing RIL‐175 mouse CCA cell line in mice treated with vehicle, CD8‐depleting antibody, or isotype control (n=6). Data are presented as mean ± SD. I) Representative immunohistochemistry (IHC) images showing CD8⁺ T cell infiltration in spleens and tumors from vehicle control, IgG, and CD8‐depleted mice. CD8 depletion efficiency is evident by the abolition of CD8⁺ staining in the αCD8 group. P‐values were calculated by two‐way ANOVA (B, H) or one‐way ANOVA (C‐G). ns=no significance, ^**^ *P* <0.01, ^***^ *P* <0.001, ^****^ *P* <0.0001.

A previous study has shown that IL‐15 suppresses tumor growth in a toxin‐induced HCC mouse model.^[^ ^43^ ^]^ Interestingly, previous studies have suggested that elevated expression of CCL17 is associated with poor prognosis in HCC.^[^ ^44^, ^45^, ^46^ ^]^ To characterize the functions of IL‐15 and CCL17 in vivo in a CCA mouse model, we expressed mouse IL‐15 or CCL17 using a lentiviral vector in a syngeneic Ras‐driven CCA cell line, RIL‐175, derived from liver tumors formed in C57BL/6 by intrasplenic injection of fetal p53^−/−^ hepatoblasts transduced with retroviral Hras^v12^ vectors.^[^ ^48^ ^]^ We transduced RIL‐175 with lentiviral vectors encoding mouse IL‐15 or CCL17 cDNA or a control empty lentiviral vector (**Figure** **4**A). RT‐qPCR analyses and cytokine arrays confirmed that IL‐15 and CCL17 were overexpressed and secreted in each cell line compared to the control (Figure S9A–D, Supporting Information). Overexpression of IL‐15 or CCL17 did not affect cell growth in vitro (Figure S9E, Supporting Information). We subcutaneously injected IL‐15‐expressing, CCL17‐expressing, or control RIL‐175 cells into immunocompetent C57BL/6 mice to initiate a syngeneic transplant mouse model of CCA. To confirm the expression and secretion of IL‐15 and CCL17 in vivo, we conducted cytokine array analysis of tumor lysates. The result showed strong IL‐15 and CCL17 expression in xenograft tumors compared with control tumors (Figure S9F, Supporting Information). Compared to the control RIL‐175 cells, overexpression of CCL17 delayed tumor progression; moreover, overexpression of IL‐15 resulted in tumor regression in the Ras‐driven CCA transplant model (Figure 4B). We harvested subcutaneous tumors 13 days after transplantation, and measured tumor volume in control tumors (735.4±227.8 mm^3^), IL‐15‐overexpressed tumors (60.5±28.9 mm^3^), and CCL17‐overexpressed tumors (295.7±141.5 mm^3^), again highlighting their critical role in delaying CCA tumor growth (Figure 4C; Figure S10, Supporting Information). These results indicate that IL‐15 and CCL17 suppress CCA tumor growth in vivo in a syngeneic transplant model.

To characterize immune cell infiltration and activation triggered by IL‐15 or CCL17, we conducted flow cytometry to measure the immune cell types in subcutaneous tumors 13 days after transplantation in the syngeneic CCA transplant model. Tumors that overexpress CCL17 or IL‐15 triggered a greater influx of CD45^+^ immune cells (Figure S11A, Supporting Information). There was an increased infiltration of CD8^+^ T cells in IL‐15‐overexpressing CCA xenograft tumors, and to a lesser (but still significant) extent in CCL17‐overexpressing tumors (Figure 4D). Either IL‐15 or CCL17 overexpression increased the level of activated CD8^+^ T cells (CD69‐positive) (Figure 4E). CD8^+^ T cells activated by IL‐15 displayed elevated levels of tumor necrosis factor alpha (TNFα), granzyme B (GZMB), and interferon gamma (IFNγ) (Figure 4F; Figure S11B,C, Supporting Information), indicating the activation of T lymphocytes. However, CCL17‐activated CD8^+^ T cells only displayed increased expression level of TNFα (Figure 4F; Figure S11B,C, Supporting Information). Moreover, CD8^+^ T lymphocytes activated by IL‐15 exhibited a larger population of memory T cells, as assessed by staining of CD62L^+^ and CD44^+^ surface markers (Figure 4G).

Since IL‐15 is known to stimulate both CD8⁺ T cells and NK cells, we next examined whether NK cells were also activated in IL‐15/CCL17‐overexpressing tumors. Flow cytometry analysis showed increased NK cell frequency and activation markers (NK1.1, CD69, TNFα, IFNγ, and Granzyme B) in IL‐15 overexpressing tumors compared with controls (Figure S11D–H, Supporting Information), indicating enhanced innate immune activity. Although NK cell abundance was not elevated in CCL17 overexpressing tumors, there was a slight increase in activated NK cells, shown by maker CD69, TNFα, and IFNγ (Figure S11D–H, Supporting Information).

To determine whether CD8⁺ T cells are functionally required for the anti‐tumor effect of IL‐15, we performed CD8⁺ T cell depletion experiments using anti‐CD8α antibody in the RIL‐175 xenograft model. As shown in Figure 4H, depletion of CD8⁺ T cells completely abolished the tumor regression observed in IL‐15‐overexpressing tumors. Immunohistochemistry of spleen and tumor sections confirmed efficient CD8⁺ T cell depletion (Figure 4I). These results demonstrate that the increased CD8⁺ T cell infiltration is not merely correlative but essential for IL‐15‐induced tumor regression. Collectively, these findings indicate that the increased infiltration and activation of CD8⁺ T cells is functionally required for IL‐15‐driven tumor regression, whereas CCL17 exerts a more modest effect.

### 2.7 IL‐15 Expression Blocks the Progression of CCA Liver Tumor in the TKP Model

**Figure 5:**
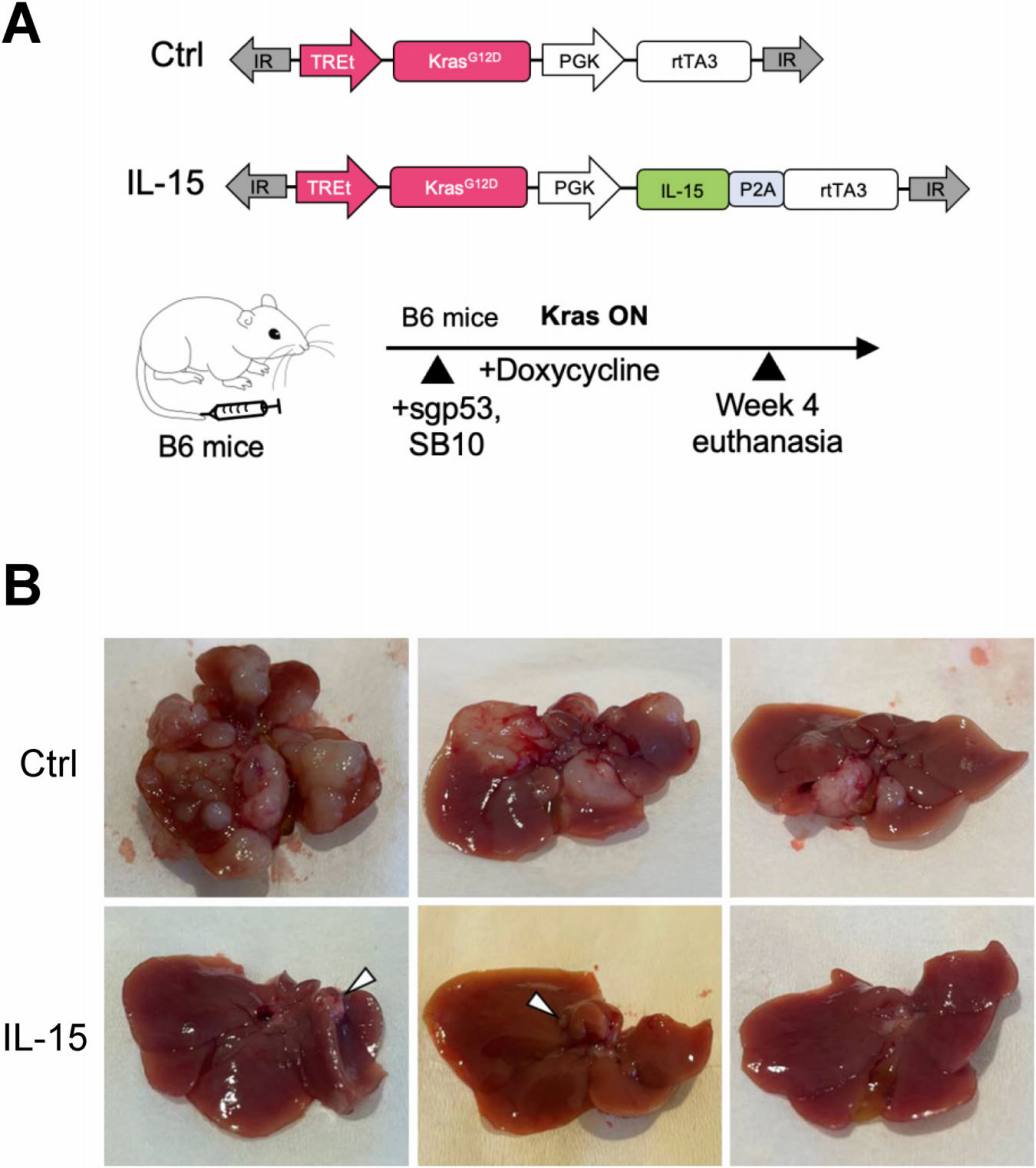
IL‐15 suppressed tumor formation in the TKP CCA mouse model. A) IL‐15‐p2A was cloned into the TRE.*Kras* transposon plasmid and injected with transposase and sgp53‐Cas9 plasmids into B6 mice. B) Tumor burden with and without IL‐15 expression was examined 4 weeks after injection. Arrows denote small tumors seen in the IL‐15 group (n=3 mice).

To further test the anti‐tumor effect of IL‐15 in the TKP liver model, we cloned a new transposon construct constitutively expressing mouse IL‐15: TRE‐*Kras* ^G12D^‐PGK‐*IL15*‐rtTA (**Figure** **5**A). We co‐delivered plasmids expressing SB transposon, transposase, and Cas9 nuclease with sgp53 into the mouse liver by hydrodynamic tail‐vein injection. In the TKP CCA model, IL‐15 had a strong anti‐tumor effect, demonstrated by a remarkable difference between surface tumor numbers of the IL‐15 group and the control group (Figure 5B). These results provided further validation of the anti‐tumor effect and therapeutic potential of IL‐15 in CCA.

### 2.8 *KRAS* ^G12D^ Inhibitor MRTX1133 Induces Senescence and IL‐15/CCL17 Expression in TKP ON Cells

**Figure 6:**
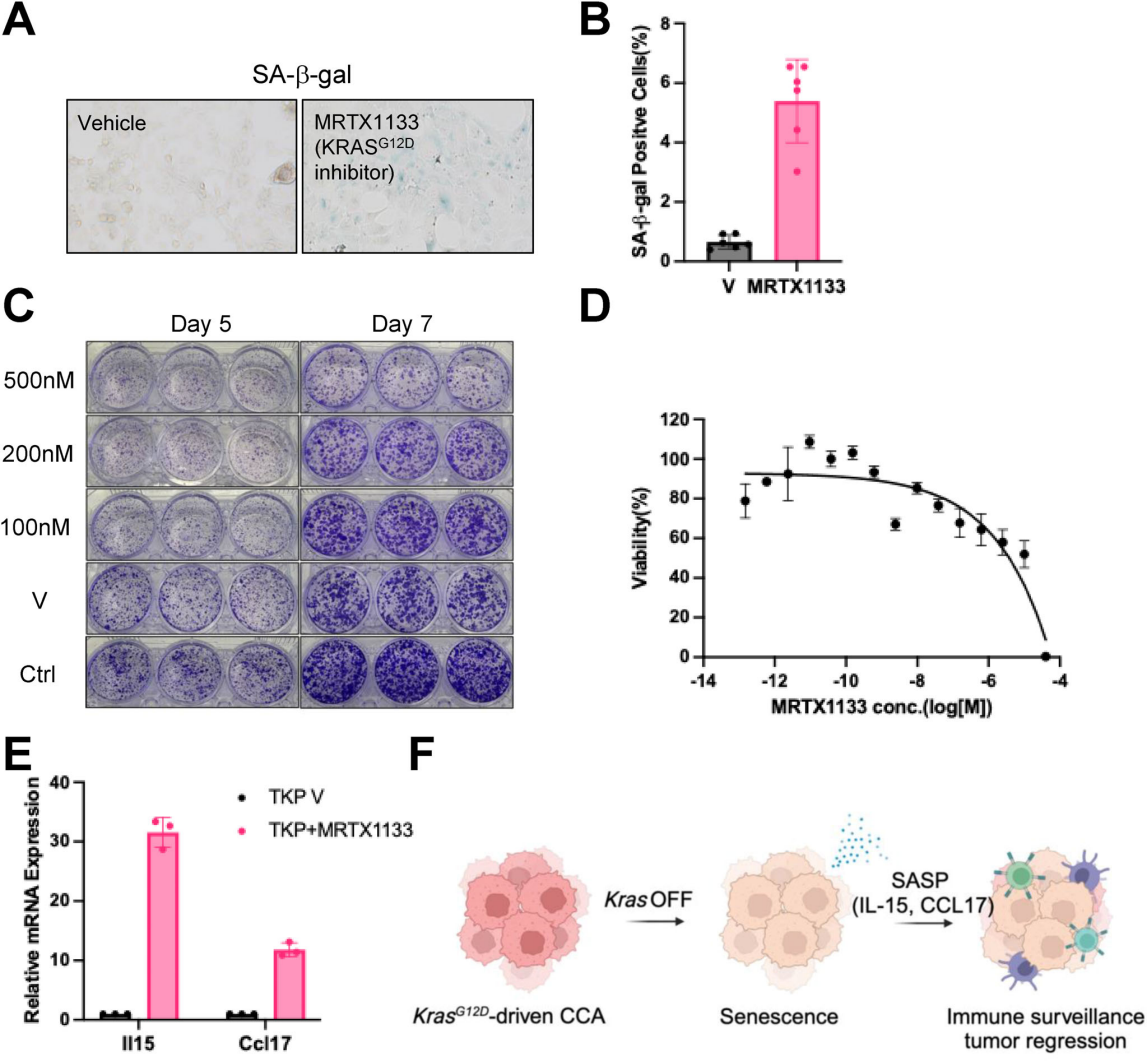
KRAS^G12D^ inhibitor MRTX1133 induces senescence and IL‐15/CCL17 upregulation. A) SA‐β‐gal staining of TKP cell line on Dox or on Dox + KRAS^G12D^ inhibitor MRTX1133 (72h). B) Quantification of (A). V, vehicle. The average is 6 of 10X fields. Data are presented as mean ± SD. C) Clonogenic assay of TKP cells (*Kras* ON) treated with MRTX1133 at day 5 and day 7. V=Vehicle (0.1% DMSO), Ctrl=control (n=3). Final concentration of DMSO in 100nM, 200nM, and 500nM is also 0.1% DMSO. D) Dose–response curve of TKP cells treated with increasing concentrations of MRTX1133 showing dose‐dependent inhibition of cell viability. Data are presented as mean ± SD (n=3). E) qRT‐PCR analysis of IL‐15 and CCL17 mRNA expression in TKP cell line treated with 500nM MRTX1133. Each group has three technical replicates. Data are presented as mean ± SD. F) Model of CCA tumor regression induced by *Kras* ^G12D^ withdrawal.

To test the pharmacological inhibition of Kras^G12D^, we treated TKP cells with a KRAS^G12D^ inhibitor, MRTX1133.^[^ ^11^ ^]^ The inhibitor induced cellular senescence and reduced cell viability in a dose‐dependent manner at different timepoints (**Figure** **6**A–D). MRTX1133 also upregulated IL‐15 and CCL17, as demonstrated by qRT‐PCR (Figure 6E), which was consistent with *Kras* withdrawal. Together, these results provided preclinical validation that the KRAS^G12D^ inhibitor induces senescence and increases expression of IL‐15 and CCL17.

## 3 Discussion

Our study established an in vivo model to study the mechanisms by which mutations in oncogenic *KRAS* contribute to CCA tumor maintenance. Conditional withdrawal of oncogenic *Kras* led to significant immune‐mediated tumor regression, highlighting the critical role of *Kras* in maintaining CCA tumors. Despite being driven by both oncogenic *Kras* and loss of *p53*, CCA tumors were sensitive to the withdrawal of *Kras^G12D^* even in the absence of tumor suppressor *p53*. This finding provides preclinical evidence for investigating KRAS‐targeted therapies as a potential treatment for CCA, which usually progresses rapidly and is often diagnosed in an unresectable stage. This mouse model will be valuable in testing *Kras* ^G12D^ inhibitors alone or in combination with immune‐checkpoint therapies for CCA.

We propose that oncogenic *Kras* withdrawal results in cell cycle arrest through p53‐independent senescence. Previous studies have demonstrated that *Kras^G12D^* withdrawal induces tumor regression in a lung adenocarcinoma mouse model and PDAC mouse models through apoptosis.^[^ ^20^, ^21^, ^22^ ^]^ By contrast, cellular senescence does not induce cell death but rather a stable, terminal arrest of proliferation.^[^ ^49^ ^]^ However, senescent cells can induce a non‐cell autonomous program known as the SASP that can trigger immune surveillance.^[^ ^7^ ^]^ We propose that oncogenic *Kras* withdrawal results in activation of TGF‐β and SMAD signaling pathways, elevated expression of cell cycle inhibitors, and senescence. While TGF‐β activation was observed during senescence, whether this pathway is required for senescence induction remains to be determined. Future studies using pharmacological or genetic inhibition of TGF‐β signaling will help clarify its mechanistic role. The senescent cells secrete proinflammatory factors, such as IL‐15 and CCL17, to recruit and activate CD8^+^ T cells for tumor clearance (Figure 6E). Consistently, recent studies have shown that the Kras^G12D^ inhibitor induces infiltration of neutrophils and CD8^+^ T cells in pancreatic tumor models.^[^ ^50^, ^51^, ^52^ ^]^ Our scRNA‐Seq results revealed significant enrichment of T cells, dendritic cells, B cells, vascular cells, and CAFs in the *Kras* OFF TKP tumor. However, one limitation of our study is that the single‐cell RNA‐seq analysis was performed on only one *Kras* ON and one *Kras* OFF tumor sample. While these data provided valuable insights into CD8⁺ T cell infiltration and other immune cell populations, the small sample size may not fully account for inter‐individual variability, and replication in additional tumors would strengthen the generalizability of these findings. Future studies will have more biological replicates and investigate the roles these cell subpopulations play in tumor regression following *Kras* withdrawal.

Our data demonstrated that both IL‐15 and CCL17 can recruit and activate CD8^+^ T cells in the CCA tumor, perhaps through different pathways. IL‐15, but not CCL17, promoted maturation of T cells into memory T cells. Our result of IL‐15 is consistent with previous findings, which show that hyper‐IL‐15 suppresses HCC tumor progression by promoting CD8^+^ T cells.^[^ ^43^ ^]^ Interestingly, another finding has suggested that IL‐15 enhances NK cell cytotoxicity and induces expression of perforin and granzyme B.^[^ ^53^ ^]^ Future studies may explore the role of IL‐15 in enhancing NK cell‐mediated anti‐tumor immunity in CCA, potentially expanding its therapeutic application beyond CD8⁺ T cells. Our functional studies demonstrated an anti‐tumor role of CCL17 in our CCA transplant model. However, this finding contrasts with previous reports linking CCL17 expression to poor prognosis in HCC, and this apparent discrepancy likely reflects context‐dependent effects of cytokines across different tumor types, which can be influenced by the composition of the tumor microenvironment, immune cell infiltration, and underlying oncogenic signaling pathways.^[^ ^44^, ^45^, ^46^ ^]^ Also, both IL‐15 and CCL17 did not trigger F4/80+ macrophage infiltration in xenograft CCA tumors (Figure S11I, Supporting Information). One potential drawback is that cytokine overexpression results in high levels of protein, which may not reflect normal physiological conditions. It nevertheless provides insight into cytokine/chemokine functions in CCA, which may help the prognosis process in clinics.

## 4 Conclusion

In summary, this study establishes a conditional CCA mouse model and provides functional evidence that oncogenic *Kras* withdrawal drives senescence and immune clearance, underscoring KRAS inhibition as a promising therapeutic strategy for CCA patients. Future work should test KRAS inhibitors in CCA mouse models to further validate KRAS‐targeted therapy as a potential therapeutic approach for CCA.

## 5 Experimental Section

### Animals and the TKP Model

All animal protocols were approved by the UMass Chan Medical School Institutional Animal Care and Use Committee (IACUC). Mice were kept in specific pathogen‐free conditions with access to food and water. The housing environment was controlled with a 12‐h light‐dark cycle, with lights on from 07:00 to 19:00, a temperature range of 20–26 °C, and humidity maintained between 30% and 70%. To make a TRE.KrasG12D/p53 knockout (TKP) CCA mouse model, 10 µg SB TREt‐KrasG12D‐PGK‐rtTA3; 20 µg CMV‐Cas9‐sgp53^[^ ^54^ ^]^; and 2 µg CMV‐SB10 transposase were delivered to 6–12‐week‐old C57BL/6 mice by hydrodynamic tail vein injection. To make a TKP model with a luciferase reporter, 15 µg SB TREt‐Kras^G12D^‐PGK‐rtTA3; 20 µg CMV‐Cas9‐sgp53^[^ ^54^ ^]^; and 6 µg PT2‐C‐Luc‐PGK‐SB13 transposase (Addgene 20207) were delivered to 6–12‐week‐old C57BL/6 mice by hydrodynamic tail vein injection. Mouse strain C57BL/6 was purchased from Jackson Laboratory (Bar Harbor, Maine). Plasmid DNAs were prepared with the EndoFree Maxiprep DNA Kit (Qiagen). Mice were fed on Doxycycline Diet 625 (Evigo) to induce the expression of Kras^G12D^. To inactivate Kras^G12D^ expression, mice were withdrawn from the doxycycline diet and supplied with a normal rodent diet. Four to five weeks after injection, mice were humanely euthanized by CO_2_ asphyxiation. Mouse livers were fixed in 10% (v/v) formalin overnight and dehydrated in 70% ethanol.

### Establishment of a Cell Line from a Solid Tumor

Fresh tumor tissues were obtained from the TKP CCA mouse model (Kras ON) and minced into small pieces with autoclaved dissection tools. Tumors were then enzymatically dissociated in dispase medium containing 1.3 mg mL^−1^ dispase II (Invitrogen) and 20 mm HEPE in Dulbecco's Modification of Eagle's Medium (DMEM) (Corning) for 45 min at 37 °C. The resulting cell suspension was vortexed and centrifuged at 300 × g for 5 min. The pellets were then resuspended in fresh complete growth medium [DMEM with 10% FBS (vol/vol) and 1% penicillin–streptomycin (vol/vol)], plated in 10cm^2^ tissue culture dishes, and incubated at 37 °C in 5% CO_2_ tissue culture incubator. Medium was changed every 2–3 days until cells reached confluence, after which they were passaged and expanded to establish the cell line. Mycoplasma testing was performed with MycoAlert Mycoplasma Detection Kit (Lonza) to ensure contamination‐free cultures.

### Cell Culture

Cells were cultured in DMEM, 10% serum (vol/vol), and 1% penicillin/streptomycin (vol/vol) under standard conditions, 37 °C in 5% CO_2_ tissue culture incubator. The RIL‐175 cell line is from Wen Xue.^[^ ^48^ ^]^ To induce Kras^G12D^ expression (Kras ON), the TKP cell line was cultured in complete medium (DMEM+10%FBS+1%penicillin–streptomycin) with 1 µg mL^−1^ doxycycline (Thermo Fisher). Kras OFF TKP cells were cultured with complete medium without doxycycline. All cell lines used were tested negative with MycoAlert Mycoplasma Detection Kit (Lonza).

### *KRAS* ^G12D^ Inhibition with MRTX1133

Kras ON TKP Cells were treated with KRAS^G12D^ inhibitor MRTX1133 (medchemexpress, HY‐134813) at a final concentration of 100, 200, and 500 nm for 5 and 7 days. The inhibitor was dissolved in DMSO, and the same volume of vehicle alone was used as a control. Treatment was under standard culture conditions prior to colony formation assay, SA‐β‐gal, and RNA extraction. To generate dose response curves in response to small molecule mutant Kras inhibition, Kras ON TKP cells were plated in 96‐well plates (Corning #3603) at a density of 5,000 cells per well one day prior to treatment. One day later, the media was replaced with complete medium + 1µg mL^−1^ doxycycline with escalating concentrations of MRTX1133 or vehicle (0.1% DMSO). After 5 days, cell growth was measured using the Cell‐Titer Glo Assay (Promega) according to the manufacturer's protocol.

### Colony Formation Assay

Kras ON TKP cells were cultured in complete medium with 1 µg mL^−1^ doxycycline, and Kras OFF cells were cultured in complete medium without doxycycline. After 8 days, Kras ON TKP cells were trypsinized and replated at 3,000 cells per well into 6‐well plates in triplicate, with doxycycline added to maintain Kras expression (Kras ON + Dox). Kras OFF TKP cells were also trypsinized and replated under the same conditions. In the Kras OFF ‐Dox group, doxycycline was withdrawn, while in the Kras OFF +Dox group, doxycycline was reintroduced to the culture medium. After 7–14 days, cells were fixed with 0.5% glutaraldehyde and stained with 0.5% crystal violet. The cells were then washed with water and dried at room temperature. For colony formation assay following KRAS^G12D^ inhibition with MRTX1133, cells were plated at 2,000 cells per well in 6‐well plates. Cells were fixed and stained after inhibitor treatment.

### Senescence‐Associated Beta‐Galactosidase Assay

SA‐β‐gal staining was conducted according to established protocols at pH 5.5 for mouse cells and tissues as described.^[^ ^40^, ^41^, ^55^ ^]^ Fresh frozen tumor tissue sections or cells grown in 6‐well plates were fixed in 0.5% glutaraldehyde in PBS for 15 min. After fixation, the samples were rinsed with PBS containing 1 mm MgCl_2_ and then incubated in a staining solution made up of PBS with 1 mm MgCl_2_, 1 mg mL^−1^ X‐Gal, and 5 mm of both potassium ferricyanide and potassium ferrocyanide for 4–18 h. Tissue sections were counterstained with eosin.

### Histology and Immunohistochemistry

Dehydrated tissues were embedded in paraffin, cut into 4 µm sections, and stained with hematoxylin and eosin (H&E) by UMass Chan Medical School Morphology Core. For immunohistochemistry staining, tumor sections were deparaffinized with xylene and dehydrated with serial ethanol dilutions. To retrieve the antigen, tissues were boiled for 10 min with 1 mm citrate buffer at pH 6.0 (Vector Labs). 3% hydrogen peroxide was used to block endogenous peroxidase activities for 10 mins at room temperature. Tissues were then blocked with 5% normal horse serum (Vector Labs) for 2 h at room temperature and incubated with primary antibodies CK19 (EPNCIR127B; 1:400) (Abcam); CD8 (4SM15; 1:100) (Thermo); F4/80(D2S9R: 1:300) (Cell Signaling) 4°C overnight. On the second day, tissues were incubated with secondary antibodies (HRP anti‐rabbit IgG, HRP anti‐rat IgG, Vector Labs) for 2 h at room temperature, and diaminobenzidine (DAB) substrate/chromogen for development (Fisher Scientific). Slides were counterstained with hematoxylin and bluing reagent, dehydrated in ethanol and xylene, and sealed with a coverslip for long‐term storage. H&E or IHC images were captured using a Leica DMi8 microscope. IHC slides were quantified by the selection of 10 random fields per section, and positive cells per 20× field were counted.

### Immunofluorescence

Tissue sections were prepared for immunofluorescence staining using the standard protocol as described above for IHC. The following primary antibodies were used: CK19 (EPNCIR127B; 1:400) (Abcam); CD8 (4SM15; 1:100) (Thermo); CD69 (Cat# AF2386; 1:100) (R&D Systems). Primary antibodies were applied overnight. The following secondary antibodies were used: Alexa Flour anti‐Goat 594 (Cat# A32758; 1:250); anti‐Rat (Cat# A48272; 1:250); anti‐Rabbit (Cat# A21207; 1:250). The secondary antibodies were applied for 1 h at room temperature. Fluorescence antibodies‐labeled slides were mounted with Prolong Gold Antifade Mountant with DAPI (Cat# P36931; Thermo Fisher).

### Immunoblot Analysis

TKP cells were seeded in a 6‐well plate at a density of 10^6^ and allowed to adhere overnight in complete growth medium with 1 µg mL^−1^ doxycycline. Kras ON TKP cells were collected at 70–80% confluency, while Kras OFF TKP cells were collected at day 8 after doxycycline withdrawal (Kras OFF D8). TKP cells were starved before collection for immunoblot analysis. To induce starvation, complete growth medium was replaced with starvation medium (DMEM + 0.1%FBS + 1%penicillin–streptomycin) for 24 h prior to cell collection. Cells were lysed with RIPA buffer (Boston bioproducts) supplemented with protease inhibitor (Roche) and phosphatase inhibitor (Thermo Fisher). Cells were further lysed with sonication. Protein concentration was measured by the BCA assay kit (Thermo Fisher). Equal amounts of protein (≈25 µg) were loaded onto NuPAGE 4–12% Bis‐Tris Protein Gels (Invitrogen) and run at 125 V for 90 mins. Protein was transferred to polyvinylidene fluoride (PVDF) membrane and incubated with indicated antibodies: anti‐SMAD2 (5339T, CST), anti‐phospho‐SMAD2 (3108T, CST), anti‐GAPDH (MAB374, EMD), followed by incubation with distinct fluorophore‐conjugated secondary antibodies. Images were captured using the Odyssey system (Li‐Cor Biosciences).

### RNA Extraction and Quantitative Real‐Time PCR

Kras ON and Kras OFF D8 RNA was extracted with the RNeasy min kit (Qiagen). 500 ng to 1 µg of RNA was used to synthesize complementary DNA (cDNA) using the high‐capacity cDNA reverse‐transcription kit (Thermo Fisher) according to the manufacturer's protocol. Real‐time quantitative PCR analysis was performed with SsoFast EvaGreen Supermix (Bio‐Rad). Beta‐actin served as an internal control for real‐time PCR. All the primers for real‐time PCR are listed in Table S1 (Supporting Information).

### Cytokine Array

TKP cells were plated in triplicate in 6‐well plates. Conditioned media were collected from Kras ON and Kras OFF D8 TKP cells and diluted 1:2 with phosphate‐buffered saline (PBS). RIL‐175 Ras‐driven CCA cell lines were plated in duplicates in a 6‐well plate. 48 h later, Conditioned media were collected from RIL‐175 cell lines and diluted 1:2 with PBS. Aliquots (100 µL) of the conditioned media were analyzed using a multiplex immunoassay (Mouse Cytokine/Chemokine 44‐Plex array) from Eve Technologies. For the tumor lysate cytokine array, RIL‐175 Ras‐driven CCA xenograft tumors were harvested and lysed in RIPA buffer, and protein concentration was measured using the BCA assay and normalized to the sample with the lowest protein concentration.

### RNA Seq

RNA‐Seq data were aligned to GRCm38 (mm10), and an RPKM matrix of transcript counts was generated. The R packages “limma” and “edgeR” were used to normalize the transcriptomic data and identify differentially expressed genes, respectively. Specifically, genes with counts per million (CPM) ≥1 in at least 3 samples were retained for differential expression analysis to remove lowly expressed genes. Differentially expressed genes were identified with an adjusted p‐value (FDR) < 0.05 and an absolute log2 fold change > 2. Volcano plots were generated by R. The expression of senescence‐associated cell cycle and SASP genes was from Ref. [40]. A heat map of gene expression (log2 fold change, LFC) was generated in R.

### GSEA Analysis

Gene set enrichment analysis (GSEA) was performed using GSEA and R software.^[^ ^56^ ^]^ Ontology gene sets and immunologic signature gene sets were downloaded from the Human Molecular Signatures Database.^[^ ^57^ ^]^ The transcriptomic dataset used for GSEA was derived from RNA‐seq of Kras ON and Kras OFF TKP cell lines. RNA sequencing data are available upon reasonable request from the authors.

### Single‐Cell RNA Sequencing and Analysis

Tumor tissues were dissected from the liver and dissociated on the gentleMACS Tissue Dissociator (Miltenyi) using the Tumor Dissociation Kit, Mouse (Miltenyi). The dissociated cells were filtered through a 30‐µm strainer to remove debris, and red blood cells were lysed using RBC lysis buffer (Miltenyi). An aliquot of cell suspension was stained with Trypan blue, and viable cells were counted on the Cellometer Auto T4 Brightfield Cell Counter (Nexcelom). 10 000 viable cells were loaded onto the Chromium Controller (10× Genomics) using the Chromium Next GEM Single Cell 3' Kit (10x Genomics). Library construction was performed following the manufacturer's standard protocol (10× Genomics). The resulting libraries were sequenced on an Illumina NovaSeq X Plus platform. Raw sequencing data were processed using Cell Ranger v7.0 (10× Genomics)^[^ ^58^ ^]^ and aligned to the mouse genome (mm39), with the KrasG12D‐rtTA3 transgene added to assemble the reference genome. Subsequent analysis was performed using Seurat v5.0.^[^ ^59^, ^60^ ^]^ Quality control excluded cells with fewer than 200 detected genes or more than 10% mitochondrial gene content. The integrated dataset was normalized using the LogNormalize method. Datasets were integrated using Seurat's CCA‐based integration.^[^ ^60^ ^]^ The top 2,000 highly variable genes were identified by the Variance Stabilizing Transformation (VST) method^[^ ^61^ ^]^ and used for principal component analysis (PCA). Cells were clustered using the Louvain algorithm^[^ ^62^ ^]^ with a resolution of 0.5, based on the top 10 principal components, resulting in 15 subclusters across 13,329 cells. Clusters were visualized using Uniform Manifold Approximation and Projection.^[^ ^63^ ^]^ Cell types were assigned to clusters based on the expression of known marker genes: immune cells (Ptprc), macrophages (Csf1r, C1qa, Cd68, Ccr2), neutrophils (S100a8, Cxcr2), T cells (Cd3d, Cd3e), vascular cells (Pecam1, Flt1), dendritic cells (Xcr1, Cd209a), tumor cells (Krt8, Krt18, rtTA3), cancer‐ associated fibroblasts (Dcn, Col1a1, Acta2), and B cells (Cd79a, Cd19, Igkc). To assess the impact of KrasG12D withdrawal, the proportions of each cell type were calculated for Kras ON and Kras OFF tumors and compared using a Fisher's exact test (FET) to determine enrichment. Within the T cell cluster, subpopulations were identified based on the expression of Cd8a (CD8+ T cells) and Cd69 (CD69+ T cells), including CD8+ CD69+ double‐positive cells. The enrichment of these T cell subpopulations in Kras OFF vs Kras ON tumors was similarly evaluated using an FET. Ligand‐Receptor interaction scores were calculated for relevant clusters as the product of the mean CPM for the ligand in the sender cluster and the receptor in the receiver cluster, with a detection threshold >0.1 CPM to account for dropout. Only pairs with scores greater than 20 in at least one condition were prioritized for downstream analysis. Scores were scaled ×10 for visualization. Pathway enrichment analysis was performed on DE marker genes per receiver cluster to assess downstream effects of prioritized L‐R pairs, comparing inferred activity in Kras‐ON vs Kras‐OFF. Gene sets were sourced from the KEGG and Reactome databases. Enrichment was calculated using hypergeometric tests in Python (scipy.stats v1.11.4), with the equation:

(1) $$\begin{array}{ccc} p & = & 1-\sum_{i=0}^{k-1}(\mathrm{Ki})(N-\mathrm{Kn}-i)(\mathrm{Nn})\;p\\ & = & 1-\sum_{i=0}^{k-1}\;\setminus \mathrm{frac}\{\setminus \mathrm{binom}\left\{K\right\}\left\{i\right\}\;\setminus \mathrm{binom}\left\{N-K\right\}\left\{n-i\right\}\}\\ & & \times \;\{\setminus \mathrm{binom}\{N\}\{n\}\}\;p\\ & = & 1-\sum i=0k-1(\mathrm{nN})(\mathrm{iK})(n-\mathrm{iN}-K) \end{array}$$

where N is the background gene universe (≈20 000 protein‐coding mouse genes from Ensembl GRCm39), K is the pathway gene set size, n is the number of cluster markers, and k is the overlap count. Overlaps were determined by matching marker genes to pathway sets. Small clusters with *n* < 50 were pooled (Clusters 9+13) to improve statistical power. Enrichment results were visualized as a horizontal bar plot using Matplotlib (v3.8.0) in Python, with the bar length representing the number of overlapping genes and the color intensity representing the ‐log10(p‐value) for significance scaling. Clusters were annotated on the left, and exact p‐values were labeled on each bar. Statistical thresholds (e.g., p < 0.05) were applied for multiple testing.

### IL‐15 and CCL17 Overexpression with Lentivirus

Murine IL‐15 and CCL17 *c*DNA was isolated from a mouse cDNA library and cloned into a lentiviral vector with EF1αpromoter. Lentiviruses were packaged by co‐transfection of 293FS cells with expression constructs (IL‐15, CCL17, or Empty) envelope vectors (VSV‐G), and packaging plasmids (Delta 8.2) using polybrene. RIL‐175 cell lines were transduced with IL‐15, CCL17, or control Empty constructs, and cell selection was performed with 500 µg mL^−1^ hygromycin for 1–2 weeks. IL‐15 and CCL17 overexpression was confirmed by qRT‐PCR and cytokine array.

### RIL‐175 CCA Xenograft Model

RIL‐175 control, IL‐15, and CCL17 expressed cell lines were harvested for implantation when they reached 70–80% confluency. Prior to implantation, cells were trypsinized, resuspended in PBS, and mixed 1:1 ratio with Matrigel (Thermo Fisher) to a final volume of 200 µL per injection. Six‐week‐old female C57BL/6 mice were purchased from Jackson Laboratory (Bar Harbor, ME). C57BL/6 mice were randomly allocated into the control group, IL‐15 group, and CCL17 group. For xenograft establishment, mice were anesthetized with isoflurane (1–3% in oxygen) and injected subcutaneously into both the right flank and left flank with 200 µL of the cell suspension (1 × 10^6^ cells). Tumor sizes were measured with digital calipers and calculated by the equation: $Volume=\frac{\pi \;\left(Length\right)\left(Width^{2}\right)}{6}$ daily. RIL‐175 xenograft tumors were harvested at day 13 after injection for further analysis.

### Flow Cytometry Analysis

To prepare single cell suspensions for flow cytometry analysis, RIL‐175 xenograft tumors were minced with scissors into small pieces and placed in 5 mL of collagenase buffer (1× HBSS with calcium and magnesium (Gibco), 1 mg mL^−1^ Collagenase V (Sigma–Aldrich), and 0.1 mg mL^−1^ DNase I) in C tubes and then processed using program 37C_m_TDK1_1 on a gentleMACS Octo dissociator with heaters (Miltenyi Biotec). Spleen was placed in 3 mL of PBS with 2% FBS in C tubes and dissociated using program m_spleen_01 on a gentleMACS Octo dissociator with heaters (Miltenyi Biotec). Dissociated tissue was passed through a 70 µm cell strainer and centrifuged at 500 × g for 5 min. To remove red blood cells, samples were lysed with ACK lysis buffer (Quality Biological) for 5 min and centrifuged, and resuspended in PBS with 2% FBS. Then, samples were blocked with anti‐CD16/32 (FC block, BD Pharmigen) for 20 min and then incubated with the following antibodies for 30 min on ice: CD45 AF700 (30‐F11; 1:320), NK1.1 BV605 (PK136; 1:200), CD3 BV650 (17A2; 1:300), CD8 PE‐Cy7 (53‐6.7; 1:400), CD62L PE‐Dazzle 594 (MEL‐14; 1:200), CD44 BV785 (IM7; 1:100), CD69 APC‐Cy7 (H1.2F3; 1:200), F4/80 APC (BM8; 1:200) (Biolegend). DAPI was used to distinguish live/dead cells. Flow cytometry was performed on a BD FACSymphony A5. Data was collected using BD FACSDiva Software, version 8.0, and analyzed using FlowJo, version 10.8.1 (TreeStar).

For analysis of Granzyme B (GZMB), Interferon‐gamma (INFγ), and Tumor Necrosis Factor‐alpha (TNFα) expression in NK and T cells, dissociated tumor tissue was resuspended in RPMI media with 10% FBS and 1% penicillin‐streptomycin and incubated for 4 h with PMA (20 ng mL^−1^, Sigma–Aldrich), Ionomycin (1 µg mL^−1^, STEMCELL technologies), and monensin (2 µm, Biolegend) in a humidified incubator at 37 °C with 5% CO2. Cell surface staining was first performed with CD45 AF700 (30‐F11; 1:320), NK1.1 BV605 (PK136; 1:200), CD3 BV650 (17A2; 1:300), CD8 APC‐Cy7 (53‐6.7; 1:200) (Biolegend). For intracellular staining, samples were then fixed, permeabilized using the Foxp3/transcription factor staining buffer set (eBioscience), and then stained with GZMB APC (GB11, Biolegend; 1:100), INFγ V450 (XMG 1.2; 1:100), and TNFα PE‐Cy7 (MP6‐XT22; 1:100). GZMB, INFγ, and TNFα expression was evaluated by gating on CD3−NK1.1+ NK cells and CD3+CD8+ T cells on an BD LSR II flow cytometer as described above.

### IVIS Bioluminescent Imaging

At the indicated time post‐injection, mice were given 200 µL luciferin (Perkin Elmer) (15 mg mL^−1^) intraperitoneally. Mice were then anesthetized with 3–5 min of 1–3% isoflurane. Fifteen minutes after luciferin injection, mice were loaded into the Perkin Elmer IVIS machine for capture of luminescent signal (1‐min standard exposure, scale 5e4 to 5e5 radiance).^[^ ^64^ ^]^

### Statistical Analysis

All statistical analyses were conducted using GraphPad Prism 9 (GraphPad Software, San Diego, CA, USA). Specific statistical tests, sample sizes, and data presentation formats are described in the respective figure legends. Significance was defined as P < 0.05.

## Conflict of Interest

The authors declare no conflict of interest.

## Supporting information



Supporting Information

Supporting Information

## Data Availability

The data that support the findings of this study are available from the corresponding author upon reasonable request.
