# Supplementary material for: KRAS Withdrawal in Cholangiocarcinoma Leads to Immune Infiltration and Tumor Regression
Source: Adv Sci (Weinh). 2025 Dec 3;13(6):e11312. doi: 10.1002/advs.202511312 (PMC12866855; doi:10.1002/advs.202511312)
Supplement: Supplementary file 1 — Supporting Information [file ADVS-13-e11312-s001.pdf]

Supporting Information

Supporting Table S1

	qPCR_F (5'-3')	qPCR_R (5'-3')
Kras	CAAGAGCGCCTTGACGATACA	CCAAGAGACAGGTTTCTCCATC
Hmga2	TTCCACTCTCTCCTCGGTTT	CGGGAAGGAGAGACAGAGAC
Sox9	AGAAAGACCACCCCGATTACAAGT	CGGCGGACCCTGAGATTG
p15	CAGGCCTTCCAAAACTTGAA	CTTCAAGGTGAGGCTCAAGG
p16	CCGCTGCAGACAGACTGG	GGGGTACGACCGAAAGAGTT
p27	AAGGGCCAACAGAACAGAAG	GGATGTCCATTCAATGGAGTC
Il15	CGTGCTCTACCTTGCAAACA	TCTCCTCCAGCTCCTCACAT
Ccl17	ATGAGGTCACTTCAGATGC	GCACTCTCGGCCTACATTGG

Supporting Table S1. Forward and reverse primer sequences used for qPCR.
